# Trust and acceptance of a virtual psychiatric interview between embodied conversational agents and outpatients

**DOI:** 10.1038/s41746-019-0213-y

**Published:** 2020-01-07

**Authors:** Pierre Philip, Lucile Dupuy, Marc Auriacombe, Fushia Serre, Etienne de Sevin, Alain Sauteraud, Jean-Arthur Micoulaud-Franchi

**Affiliations:** 1 University of Bordeaux, Bordeaux, France; 2 Clinique du Sommeil, CHU de Bordeaux, Bordeaux, France; 3 SANPSY, CNRS USR 3413, Bordeaux, France; 4 Pôle addictologie, CH Charles Perrens and Unité de Soins Complexes d’addictologie (USCA) CHU de Bordeaux, Bordeaux, France

**Keywords:** Preclinical research, Developing world, Information technology

## Abstract

Virtual agents have demonstrated their ability to conduct clinical interviews. However, the factors influencing patients’ engagement with these agents have not yet been assessed. The objective of this study is to assess in outpatients the trust and acceptance of virtual agents performing medical interviews and to explore their influence on outpatients’ engagement. In all, 318 outpatients were enroled. The agent was perceived as trustworthy and well accepted by the patients, confirming the good engagement of patients in the interaction. Older and less-educated patients accepted the virtual medical agent (VMA) more than younger and well-educated ones. Credibility of the agent appeared to main dimension, enabling engaged and non-engaged outpatients to be classified. Our results show a high rate of engagement with the virtual agent that was mainly related to high trust and acceptance of the agent. These results open new paths for the future use of VMAs in medicine.

## Introduction

Mental disorders are chronic conditions requiring repetitive and time-consuming psychiatric consultations. Because of their increasing prevalence in Western societies and the shortage of physicians, there is a need for innovative clinical solutions to interview patients without mobilising human resources.^1^ Digital medicine is a promising solution to conduct patients’ follow-up, and virtual medical agents (VMAs) have been considered as potential candidates to assist real physicians.^2–4^ VMAs are embodied conversational agents (ECAs), defined in the human–computer interface (HCI) field as computer characters able to engage in face-to-face dialogue through verbal and nonverbal behaviour.^5^ ECAs have already been used in medicine for the diagnosis,^6–8^ prevention,^9,10^ and treatment of medical conditions.^11,12^ Taken together, the above studies tend to show that ECAs have a positive effect, as they rely on a virtual face-to-face interaction, thus creating empathy for users and facilitating disclosure of negatively connoted symptoms such as addiction, depression, and post-traumatic stress disorder manifestations.^6–8^ In addition, ECAs can significantly help healthcare delivery by saving physicians’ time, diminishing the variability between professional interventions, providing 24/7 and easy-to-access support and monitoring of patients, and by managing huge volumes of medical data.

Nevertheless, to achieve these medical objectives consistently over the long term, i.e., management of chronic diseases, researchers need to support patients’ engagement with VMAs^13^ by fostering a “strong doctor-patient relationship”.^14^ In a recent publication by the Lancet psychiatry commission,^14^ experts underlined two major dimensions that will consolidate engagement with health technologies: acceptance and trust. Acceptance and trust are complex and interrelated constructs evaluated with various tools in the HCI literature, and therefore need a clear theoretical background. In their article, Bhugra and colleagues suggest that acceptance can be promoted by providing easy ways to “entry and retrieval of data”, and by being “enjoyable to use”. In the HCI literature, these constructs are commonly referred to as the concepts of perceived ease of use (also called by some author usability^15^*)* and perceived usefulness (or satisfaction),^15–17^ which altogether can predict the use or rejection of technology in general^15,16^ and ECAs in particular.^18,19^

The second issue raised by Bhugra and colleagues is trust in the digital device, so that “patients feel confident in sharing their psychiatric history”,^14^ which is also a well-studied construct and a central aspect of engagement with technologies. In the HCI literature, Cassell & Bickmore^20^ proposed that trust in an ECA occurs if the user perceives credibility and benevolence in the agent. Credibility corresponds to the belief that the agent has the ability and the expertise in a specific domain to perform the task, and benevolence means that the agent cares about and will act according to one’s interest. In terms of ECA design, Cassell & Bickmore suggest that credibility can be shown to the user by using expert vocabulary, adapted appearance, or professional affiliation, whereas benevolence can be demonstrated through greetings, small talk, or references to past experiences. In a non-medical context, some studies have shown that perceived credibility and benevolence can predict acceptance of wearable technologies^21^ and ECA usage for e-commerce.^22,23^ Thus, acceptance and trust are central in the evaluation of new technologies and should be assessed systematically in the context of ECAs for medical care. Surprisingly, there is a lack of standardised and validated scales to rigorously measure these dimensions, in particular in medicine.^13^

Furthermore, engagement, acceptance, and trust do not depend only on the design of the software but may vary highly depending on the characteristics of the user. Age, gender, education, and health conditions of users can influence engagement, acceptance, and trust in technologies but have been insufficiently studied.^24,25^ In addition, the impact of the medical domain covered by the agent (i.e., type of disease targeted) on engagement, acceptance, and trust in a similar VMA has never been studied. Therefore, the characteristics of the user and the medical domain covered by the agent need to be considered when evaluating a particular technology.

Recently, our team published several articles demonstrating that ECAs can conduct reliable and valuable clinical interviews and make psychiatric diagnoses (depression and addiction) in outpatients seen in a sleep clinic.^6,7,26^ In addition, we showed that a VMA was better accepted than a questionnaire displayed on a tablet to diagnose major depressive disorder (MDD).^25^ In this new study, we explored the impact of both the characteristics of the user and the context of the psychiatric interview covered by the VMA (for depression or addiction screening) on engagement, acceptance, and trust, and attempted to determine the threshold of acceptance and trust in VMAs that are associated with positive engagement.

## Results

### Sample characteristics

A total sample of 318 patients were analysed. Patients’ characteristics are summarised in Table 1.

**Table 1:** Sample characteristics.

	Total (*N* = 318)
Age (M (SD))	45.01 (13.33)
<30 years (%)	19.9%
30–50 years (%)	38.8%
>50 years (%)	42.0%
Gender (% males)	45%
Education (in years) (M (SD))	13.36 (2.98)
Middle school (%)	10.3%
High school (%)	38.6%
University degree (%)	50.9%
Type of sleep disorder (%)	
Nocturnal breathing disorders	42.3%
Narcolepsy, hypersomnia	10.7%
Insufficient sleep syndrome	1.9%
Periodic leg movements and RLS	1.3%
Insomnia, ADHD, parasomnia	9.5%
Non-organic sleep complaints	34.4%

*RLS* restless legs syndrome, *ADHD* attention deficit hyperactivity disorder

Group comparisons showed no significant differences between patients included in Study 1 and Study 2 regarding demographic or sleep disorder characteristics (all *p* > 0.100). We therefore grouped the two populations to analyse characteristics.

Globally, participants were middle-aged (45.01 years on average; SD = 13.33), with one third aged over 50 years old. There was a slightly higher educational level than the French population average,^27^ with about half of the participants having a bachelor’s degree. Most participants suffered from nocturnal breathing disorders, which matches the prevalence among the general population.^28^ Hypersomnia and narcolepsy were also well represented, with ~ 10% of our sample suffering from these disorders. Finally, about a third of participants suffered from non-organic sleep complaints, such as asymptomatic snoring, transient insomniac sleep complaints, or sleep hygiene disorders.

### Trust, acceptance, and engagement with the VMA

**Fig. 1:**
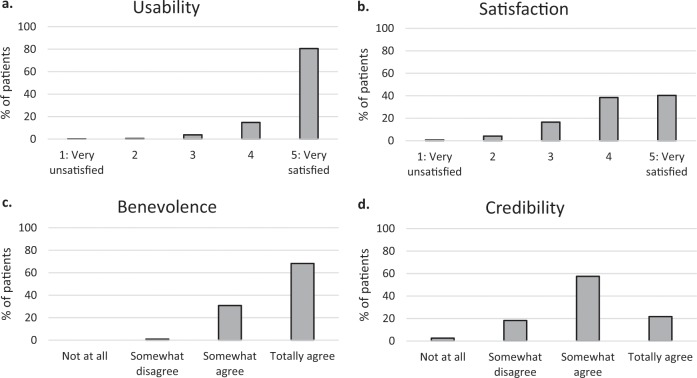
Distribution of usability, satisfaction, benevolence, and credibility perception. **a** percentage of patients’ rating for usability dimension (AES sub-score), **b** percentage of patients’ rating for satisfaction dimension (AES sub-score), **c** percentage of patients’ rating for benevolence dimension (ETQ sub-score), **d** percentage of patients’ rating for credibility dimension (ETQ sub-score).

Acceptance and trust data are presented in Fig. 1. Acceptance of the overall system (score of the AES scale) was rated very positively, with 68.2% of patients being “very satisfied” by the VMA’s usability, and 78.1% of patients rating the VMA more than three out of five for satisfaction. Regarding trust (score on the ETQ scale), the VMA was perceived as trustworthy to perform medical interviews. Indeed, 68.2% of patients “totally agreed” that the VMA was benevolent, and 79.2% of patients rated the VMA more than two out of three for credibility.

**Fig. 2:**
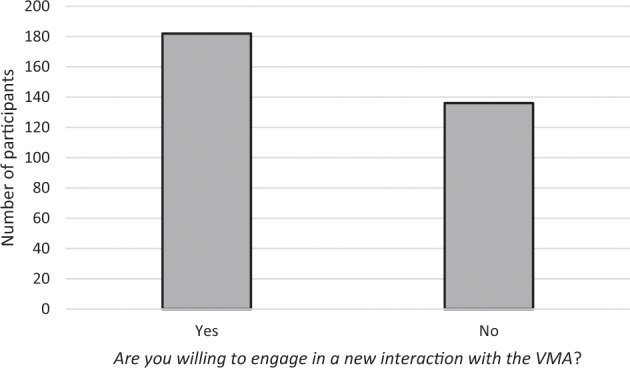
Distribution of outpatients regarding their perceived engagement with the VMA.

The distribution of patients’ answers to the Engagement question is presented in Fig. 2. More than half (57.23%) of outpatients were willing to interact with the VMA in the future, suggesting a positive engagement with the agent.

### Influence of patients’ characteristics and VMA interview on acceptance, trust, and engagement

Regarding usability sub-scores, Pearson correlation analyses revealed that age was significantly correlated with the usability sub-score of the AES, with older participants perceiving the system as easier to use than younger ones (*r* = 0.143; *p* = 0.11). Mean comparisons showed that the medical domain covered by the VMA influences usability (*t*(316) = −3.385; *p* = 0.001), and that the addiction interview is perceived as easier to use than interview screening for MDD. Other variables (education, gender, sleep disorder) remained non-significant (all *p* > 0.200), indicating that the system was perceived as easy to use irrespective of these user characteristics. Multivariate analyses (Supplementary Table 1) confirmed the significant influence of age and medical domain (F(2,316) = 8.587; *p* < 0.001).

The satisfaction sub-score was significantly correlated with age (*r* = 0.169; *p* = 0.011) and educational level (*r* = −0.179; *p* = 0.001), older and less-educated participants being more satisfied by the system than those younger and with a high level of education. However, users were satisfied by the system regardless of their gender, sleep disorder or medical domain covered by the VMA (all *p* > 0.500). Multivariate analyses (Supplementary Table 1) confirmed the significant influence of age and education (F(2, 313) = 8.915; *p* < 0.001).

Concerning trust dimensions, neither credibility sub-score nor benevolence sub-scores seemed to be influenced by any user characteristics or type of psychiatric interview conducted by the VMA. Therefore, we did not perform any multivariate analyses.

Finally, regarding engagement question (“willing to engage in a new interaction”), mean and distribution comparisons analyses showed that patients’ engagement varied with regard to VMA interview (MDD or addiction) (*χ*² (1) = 10.156; *p* = 0.001) and educational level (*t*(313) = −1.993; *p* = 0.005). The interview for depression screening, and a lower level of education were significant predictors of non-engagement with the ECA. Gender, type of sleep disorder, and age were not significantly different between engaged and non-engaged patients. Logistic regression (Supplementary Table 1) confirmed the significant influence of medical domain and education (omnibus chi-square = 15.689, df = 2, *p* < 0.001).

### Acceptance and trust thresholds associated with future engagement with the VMA

**Fig. 3:**
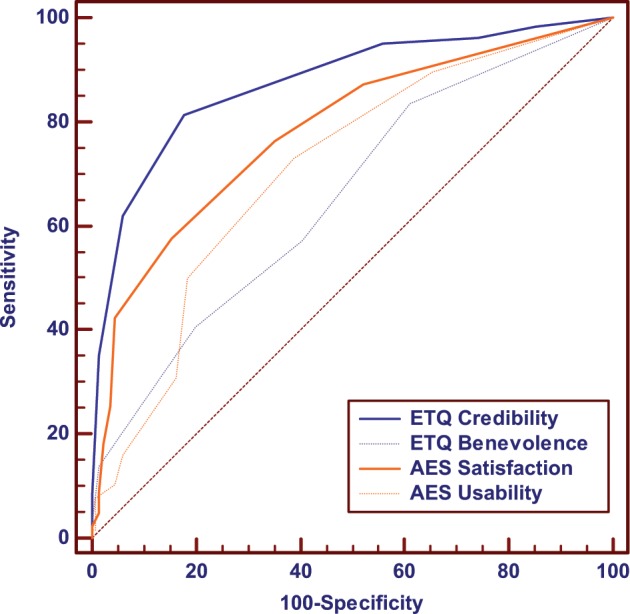
ROC curves of the four dimensions of trust and acceptance: benevolence, credibility, usability, and satisfaction, to classify engaged and non-engaged subjects.

Statistical receiver operating characteristics (ROC) analyses with the four sub-scores of acceptance and trust (usability, satisfaction, benevolence, and credibility) are presented in Fig. 3 and Supplementary Table 2.

Globally, the four scores classified patients efficiently depending on their future engagement, all area under the curve (AUC) being above 0.650. Analyses revealed cutoff scores of 13.5 and 12.5 for usability and satisfaction, respectively, and 5.5 and 8.5 for credibility and benevolence, in order to classify patients engaged with the VMA. The credibility sub-score of the ETQ obtained significantly the best classification performance compared with the other dimensions (all *p* < 0.100) and showed an AUC of 0.875 (*p* > 0.001), a cutoff sensitivity of 82.4% and a specificity of 81.3%.

## Discussion

The objective of this study was to measure engagement, and perceived acceptance and trust of VMAs used to diagnose depression and addiction disorders among outpatients. We explored the influence of outpatients’ characteristics and type of medical field covered by the VMA in the variation of engagement, acceptance, and trust. These findings are very encouraging and suggest that VMAs could help a wide range of patients. It also advocates for the consideration of user characteristics and topic of the medical intervention when designing and evaluating ECAs usage. At last, we identified acceptance and trust thresholds to quantify future engagement with the agent.

Taken together, our results show satisfactory levels of acceptance and trust independently of the gender, type of sleep disorders affecting the outpatients, and medical domain covered by the VMA. Previous studies including gender analyses of technology acceptance found contradictory results, some highlighting differences between men and women in acceptance,^19^ whereas others did not.^21^ Further studies are needed to investigate the impact of gender of the patient with regard to the gender of the VMA. There was no significant influence of user characteristics and medical domain covered by the VMA on perceived trust, but we observed significant relationships between age, level of education, and perceived acceptance of the system. Contrary to common stereotypes that older adults are less likely to adopt technologies,^29^ older patients demonstrated greater acceptance of the system displaying the medical agent. Here again, internet technologies are now widely deployed among aging populations to offset the problem of medical deserts and the loss of autonomy of seniors.^30^ In addition, several older adults felt that our VMA was like a companion who could help them to manage their health in their remotely situated houses. The public health implications are important, given the aging of the population worldwide and the growing percentage of older individuals with chronic diseases.^31^ Subjects with a low level of education were more satisfied by our system more than those with a higher level. The massive development of internet technologies in all classes of society combined with the widespread availability of “free apps” in the healthcare domain could explain these results.

Furthermore, most of our patients were willing to interact with the agent in the future, suggesting positive engagement. However, we observed that the interview for depression screening and a lower level of education seemed to favour non-engagement with the VMA. Reasons for these influences might be that the conduct of the interview varied between addiction and MDD screening, the former was based on short structured questionnaires (i.e., the CDS-5 and the CAGE questionnaire), whereas the latter involves less-structured questions (i.e., based on the DSM-5 criteria). In line with this hypothesis, our results show that the MDD interview with the VMA was perceived as less easy to use than the addiction interview. Further studies should investigate the impact of length and design of the interview on patients’ engagement. In addition, particular attention should be paid to the interaction scenario implemented by the VMA.

Results of the ROC analyses revealed threshold scores for the AES and ETQ scores and sub-scores to detect future engaged and non-engaged users in a clinical setting. This contribution could be helpful for the earlier detection of disengagement with a VMA, especially in a long-term autonomous use (e.g., at home for the follow-up of chronic patients). In particular, we show that the agent’s credibility appropriately classified > 80% of patients, which suggests that credibility is the most discriminant dimension in terms of patients’ engagement. This result confirms that VMAs can be trusted by patients if used in an appropriate clinical context.^8^ It is in line with findings showing the benefits of therapeutic alliance between patient and physicians^32,33^ and with the suggestion that alliance with digital mental health apps is crucial for the future.^13^ Credibility should therefore be a prime consideration when designing VMAs, especially for chronic disease management.

To promote the use of VMAs in clinical settings, medical and HCI experts and regulatory agencies should work together to identify and adhere to standardised vocabulary, methods for the design^34–36^ and evaluation^4,13,37^ of digital solutions for healthcare. In the longer term, this interdisciplinarity would provide the opportunity to develop VMAs fully compliant with standards and legal aspects, as stated by the International Organization for Standardization (ISO) in the standard for Human-centred design for Interactive systems (ISO 9241-210:2019)^17,38^ and by several health regulatory agencies (such as the National Institute for Health and Care Excellence,^39^ the National Health Service^40^; the Medical Research Council,^41^ or the European Medicines Agency^42^). In the academic field, among other evaluation models proposed,^43–45^ Torous and colleagues^46,47^ recently recommended four steps to be met before including new technological tools can in clinical practice: (1) consider risk and privacy issues, (2) validate efficacy for health, (3) ensure engagement, and (4) establish interoperability. By evaluating acceptance and trust in a VMA, our study conducted in the context of a psychiatric interview meets the third objective of Torous’ framework. Further studies are now needed to compare the validity of the AES and ETQ with that of other evaluation tools,^13,16,48^ whereas keeping in mind the necessity for a common and interdisciplinary vocabulary regarding engagement with technologies. At last, additional studies need to confirm the present results over repeated usage of the VMA in the patient’s home. Such studies should investigate the impact of intervention duration, frequency and learnability, severity, and evolution of clinical manifestations, the influence of health and technological representations by the patients and their professional and informal caregivers, and modalities of integration in healthcare systems, in order to promote the involvement of conversational agents for an efficient patient-centred care.

## Methods

This study follows a quantitative experimental design. Data were collected during two protocols published previously.^6,7^ In study 1,^6^ the objective was to validate the efficacy of a VMA performing MDD diagnosis. Study 2^7^ focused on the validity of the VMA to perform screening and diagnosis for tobacco and alcohol use disorders.

### Participants

Participants were recruited among outpatients seen at the Sleep Clinic at the University Hospital of Bordeaux (France) from November 2014 to May 2017. The patient population mirrored the characteristics of a patient group consulting in general practice with a common referral related to sleep complaints and a high rate of co-morbidities, including mood disorders or addiction. They were asked to participate in the study during their clinical interview by a sleep specialist. Gender, age, education, and suspected sleep disorders were collected. In all, 179 participants were recruited for Study 1 (VMA for MDD diagnosis), and 139 were included in Study 2 (VMA for tobacco and alcohol addiction screening). Patients had to be aged 18 or older, French native speakers, and have sufficient auditory and visual aptitude to interact with the VMA. A more-detailed description of the inclusion and exclusion criteria can be found in ref. ^6,7^

This project is part of a larger project on virtual reality and clinical phenotyping (PHENOVIRT) that has been approved in compliance with French and European regulations on clinical research by a local ethics committee (Comité pour la Protection des Personnes—Institutional Review Board of University of Bordeaux). All participants gave their written informed consent before entering the study.

### VMA description

In study 1, the VMA was developed to conduct a psychiatric interview in order to evaluate MDD according to the Diagnostic and Statistical Manual of Mental Disorder (DSM-5) criteria. In study 2, the agent was developed to screen for current alcohol and tobacco use disorders with an adaptation of the Cigarette Dependence Scale (CDS-5^49^) and the CAGE^50^ questionnaires. The interaction was based on a pre-determined scenario, with several options throughout the case depending on the user’s answers but leading to a single end point. The interviews were adapted by sleep specialists and computer scientists to reinforce the credibility and benevolence of the agent, notably by adding small talk and adapting the agent’s appearance. The usability of the system and satisfaction with it were considered in the design and pre-tested by the research team.

**Fig. 4:**
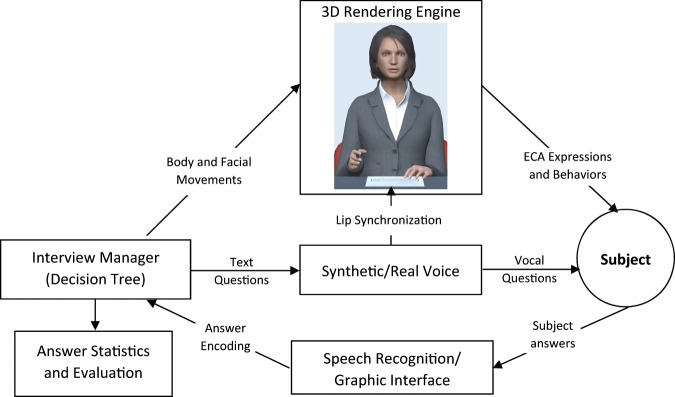
Architecture of the Embodied Conversational Agent.

Both VMAs had a female appearance were displayed on a tablet and talked to the patient with a recorded real voice. The patient could answer the VMA’s questions orally thanks to voice recognition. The virtual environment was generated by Unity 3D software (Unity-Technologies, 2014), and gestures were captured by motion capture technology. The software is based on (Fig. 4): (i) a scenario manager, based on decision trees, who coordinates the whole interview and manages the other modules, (ii) a display manager that automatically plays the voice and animations of the virtual human, (iii) an interaction manager, managing speech recognition and the graphical interface to respond to the ECA. A video of our VMA performing an interview can be found in: http://www.sanpsy.univ-bordeauxsegalen.fr/Papers/NPJ_Additional_Material.html.

### Engagement, acceptance, and trust measures

Patients’ perceptions of the VMA were quantified via two questionnaires evaluating trust in the agent and acceptance of the overall system. We used short scales to make it time-efficient and usable in clinical practice, and both questionnaires were answered directly on the tablet after interacting with the VMA.

#### Acceptance of the E-health system

To measure acceptance of the system, we used the Acceptability E-scale.^15^ This self-reported questionnaire assessing acceptance of E-health systems comprises six items (Table 2). The scale comprises a total score and two sub-scores regarding usability (i.e., the perceived ease of using the system) and satisfaction of the device (i.e., the perceived enjoyment of the use and usefulness of the system).^15,16^ Items are based on a five-point Likert scale ranging from 1: “very unsatisfied”; to 5: “very satisfied”, where higher scores indicate higher acceptance. This scale was validated in its French version^19^ in a previous publication from our team.

**Table 2:** English and French versions of the ECA Trust Questionnaire (ETQ) and the Acceptability E-scale (AES).

No.	Factor	Item	French version
AES items^15,19^			
1	Usability	How easy was the computer programme to use?	A quel point avez-vous trouvé ce programme informatique facile d’utilisation?
2	Usability	How understandable were the questions?	A quel point les questions étaient-elles compréhensibles?
3	Satisfaction	How much did you enjoy using this computer programme?	A quel point avez-vous apprécié l’utilisation de ce programme informatique?
4	Satisfaction	How helpful was this computer programme in describing your symptoms and quality of life?	A quel point ce programme informatique vous a-t-il été utile pour décrire vos symptômes et votre qualité de vie?
5	Usability	Was the amount of time it took to complete this computer programme acceptable?	Le temps consacré à répondre à ce programme informatique était-il acceptable?
6	Satisfaction	How would you rate your overall satisfaction with this computer programme?	Comment évaluez-vous votre satisfaction générale de cet outil informatique?
ETQ items			
1	Benevolence	Did you feel that your answers were correctly understood by the virtual agent?	Avez-vous eu l'impression que vos réponses ont bien été comprises par l’agent virtuel?
2	Benevolence	Did you feel that the questions asked by the virtual agent were clear?	Est-ce que les questions posées par l’agent virtuel vous ont paru claires?
3	Benevolence	Did you feel that the interview with the virtual agent was pleasant?	Avez-vous trouvé l'entretien avec l’agent virtuel agréable?
4	Credibility	Would you agree to being cared for by the virtual agent in hospital?	Seriez- vous d'accord que l’agent virtuel participe à votre prise en charge à l'hôpital?
5	Credibility	Would you agree to being cared for by the virtual agent at home?	Seriez- vous d'accord pour que l’agent virtuel participe à votre prise en charge à domicile?
6	Credibility	Did you feel that the virtual agent was competent?	Avez-vous eu l’impression que l’agent virtuel était compétent?

#### Perceived trust in the VMA

Based on the literature,^20–23^ we developed a six-item questionnaire evaluating trust in the VMA (which we named the ECA Trust Questionnaire, Table 2). Items are based on a four-point Likert scale ranging from 0: “Not at all” to 3: “Totally agree”, with a total score of 18, higher scores indicating a more favourable attitude toward the agent. The questionnaire is subdivided into two three-item sub-scores regarding perceived credibility (i.e., perception that the agent has the ability and the expertise to conduct a medical intervention) and benevolence of the agent (i.e., perception that the agent is well-intentioned and will accurately take one’s interests into account), each scored out of 9. To validate the psychometric properties of the scale, several statistical analyses were performed. First, to ensure its construct validity, we conducted a principal component analysis. Results validated breaking down the scale into two factors (KMO value: .67), Factor 1 (corresponding to credibility) grouping items 4, 5, and 6; and Factor 2 (corresponding to benevolence) grouping items 1, 2, and 3 (altogether explaining 61.19% of the variance). Varimax-rotated factors loading of the ETQ scale are presented in Supplementary Table 3. To ensure that the ETQ explores factors other than those on the AES scale, we conducted confirmatory factor analyses on the six items of the ETQ scale plus the six items of the AES scale with two latent variables underlying the four sub-scores of ETQ and AES. Root Mean Square Error of Approximation (RMSEA), Standardised Root Mean Square Residual (SRMR) and Comparative Fit Index (CFI), indicated an acceptable model fit (RMSEA = 0.104; SRMR = 0.027; and CFI = 0.988). Second, to ensure the internal consistency of the ETQ, Cronbach’s alpha coefficients were calculated. According to Cronbach’s threshold,^51^ analyses showed acceptable results (alpha = 0.71, and ranging from 0.66 to 0.71 after removing one item). Finally, floor and ceiling effects measuring the proportions of participants obtaining the lowest and the highest scores for each item were calculated to assess the distribution of responses. Obtained floor effects ranged from 0.6% to 16.0% and ceiling effects ranged from 14.5% to 61.0%. Taken together, these analyses suggest that the scale has satisfactory psychometric properties.

#### Future engagement with the VMA

To estimate patients’ willingness to stay engaged with the VMA during repetitive use, we administered one two-choice question: “Are you willing to engage in a new interaction with the virtual medical agent?” after the interview with the VMA. Patients could answer “Yes” or “No”. We refer hereafter to this question as the “Engagement question”.

### Statistical analyses

Quantitative variables were expressed with means (M), and standard deviations (SD), and qualitative variables were expressed using distributions and percentages. To investigate factors associated with acceptance (usability and satisfaction sub-scores of the AES), trust (credibility and benevolence sub-scores of the ETQ) and engagement (answer to Engagement question), we conducted univariate analyses with Pearson correlation analyses between two continuous variables (age, education, AES sub-scores, ETQ sub-scores), mean comparisons (*t* test or analysis of variances) for categorical variables (gender, type of sleep disorder, medical domain of the agent) and distribution comparisons (*χ*² tests) when both variables are categorical (when comparison is based on the Engagement question). When associations appeared significant (*p* *<* 0.05), we conducted multivariate analyses using linear regressions for AES et ETQ sub-scores as dependent variables, and using logistic regressions for Engagement question as the dependent variable.

At last, to identify acceptance and trust thresholds that would induce future patients to engage with the system, we performed ROC analyses using AES and ETQ sub-scores as parameters, and the Engagement question as binary classifier. For these analyses, AUC, sensitivity/specificity and positive/negative predictive reports were presented. A cutoff point was obtained by selecting the point on the ROC curve that maximised both sensitivity and specificity. Analyses were performed using SPSS software (version 18, PASW Statistics), RStudio (version 1.2.1335, RStudio Inc) and MedCalc (version 14.8 for Windows).

### Reporting summary

Further information on experimental design is available in the Nature Research Reporting Summary linked to this paper.

## Supplementary information

Supplementary material

Reporting summary

## Data Availability

The data that support the findings of this study are available from the corresponding author upon reasonable request.
