# Supplementary material for: Trust and acceptance of a virtual psychiatric interview between embodied conversational agents and outpatients
Source: NPJ Digit Med. 2020 Jan 7;3:2. doi: 10.1038/s41746-019-0213-y (PMC6946646; doi:10.1038/s41746-019-0213-y)
Supplement: Supplementary file 1 — Supplementary material [file 41746_2019_213_MOESM1_ESM.docx]

**Supplementary Information**

**Supplementary Table 1.** Linear and logistic regressions of factors associated with acceptance, trust and engagement with the VMA

| **AES usability – linear regression** | | | | |
| --- | --- | --- | --- | --- |
|  | **B** | **SE B** | **β** | **Significance** |
| Age  VMA domain | .019  -.753 | .009  .234 | .120  -.178 | .031  .001 |
| **AES satisfaction – linear regression** | | | | |
| Age  Education | .025  -.126 | .010  .043 | .145  -.164 | .010  .003 |
| **Engagement question – logistic regression** | | | | |
|  | **β estimate** | **SE β** | **Wald statistic** | **Significance** |
| Education  VMA domain | .080  -.803 | .040  .238 | 4.029  11.371 | .045  .001 |

*Notes.* VMA: Virtual Medical Agent; SE: Standard Error

**Supplementary Table 2.** Area under the curve with 95 percent confidence interval [CI], cut-off scores, and sensitivity and specificity for these scores, for performance of trust and acceptance variables to classify future engagement with the VMA

|  | **AUC** | **P value** | **Cut-off score** | **Cut-off**  **Sensitivity** | **Cut-off**  **Specificity** |
| --- | --- | --- | --- | --- | --- |
| **AES sub-scores**  Usability  Satisfaction  **ETQ sub-scores**  Benevolence  Credibility | .708 [.650; .766]  .782 [.731; .832]  .654 [.594; .714]  .875 [.837; .913] | < .001  < .001  < .001  < .001 | 13.5  12.5  8.5  5.5 | .610  .846  .390  .824 | .731  .577  .835  .813 |

**Supplementary Table 3:** Loading of Varimax-rotated factors of the ETQ scale

| **ETQ items** | **Component 1** | **Component 2** |
| --- | --- | --- |
| ETQ 4  ETQ 5  ETQ 6  ETQ 1  ETQ 2  ETQ 3 | **.866**  **.852**  **.568**  .103  .059  .428 | .119  .011  .250  **.812**  **.792**  **.526** |

*Notes.* Bolded values indicate that the items can be grouped in the corresponding component.
